# Multimodal audio-visual robot fusing 3D CNN and CRNN for player behavior recognition and prediction in basketball matches

**DOI:** 10.3389/fnbot.2024.1284175

**Published:** 2024-03-06

**Authors:** Haiyan Wang

**Affiliations:** School of Physical Education, Xinxiang University, Xinxiang, Henan, China

**Keywords:** multimodal, 3D CNN, CRNN, LSTM, behavior recognition, behavior prediction, basketball matches

## Abstract

**Introduction:**

Intelligent robots play a crucial role in enhancing efficiency, reducing costs, and improving safety in the logistics industry. However, traditional path planning methods often struggle to adapt to dynamic environments, leading to issues such as collisions and conflicts. This study aims to address the challenges of path planning and control for logistics robots in complex environments.

**Methods:**

The proposed method integrates information from different perception modalities to achieve more accurate path planning and obstacle avoidance control, thereby enhancing the autonomy and reliability of logistics robots. Firstly, a 3D convolutional neural network (CNN) is employed to learn the feature representation of objects in the environment for object recognition. Next, long short-term memory (LSTM) is used to model spatio-temporal features and predict the behavior and trajectory of dynamic obstacles. This enables the robot to accurately predict the future position of obstacles in complex environments, reducing collision risks. Finally, the Dijkstra algorithm is applied for path planning and control decisions to ensure the robot selects the optimal path in various scenarios.

**Results:**

Experimental results demonstrate the effectiveness of the proposed method in terms of path planning accuracy and obstacle avoidance performance. The method outperforms traditional approaches, showing significant improvements in both aspects.

**Discussion:**

The intelligent path planning and control scheme presented in this paper enhances the practicality of logistics robots in complex environments, thereby promoting efficiency and safety in the logistics industry.

## 1 Introduction

In today's fast-growing field of deep learning, multimodal data analysis and prediction has become a compelling research direction (Maimaitijiang et al., 2020). Multimodal data refers to different types of data, such as images and sounds, which often co-exist in various real-world scenarios. With the improvement of computing power and the development of data acquisition technology, utilizing multimodal data to achieve more accurate and comprehensive analysis and prediction has become an important challenge (Giannakos et al., 2019; Hosseini et al., 2020). Basketball game is a dynamic and complex sports event, and its data contains rich information. Therefore, it is of great research significance to use multi-modal data for player behavior recognition and prediction in basketball games. The application of multimodal audio-visual robot in basketball game can help to improve the understanding and analysis of the game. By analyzing video and voice data simultaneously, we can get a more complete picture of player movements, position changes, and the real-time situation of the game. This not only helps coaches, teams and spectators better understand the game process, but also provides a scientific basis for tactical formulation and decision-making. Below are some common deep learning methods used to recognize and predict behavior.

Convolutional Neural Networks (CNN) (Mohamed et al., 2020) perform well in image processing tasks, capable of extracting spatial features in images. Local and global features of images can be learned automatically through convolutional and pooling layers. In basketball behavior recognition, CNN can be used to extract players' spatial position and action features. But CNN mainly focuses on local features and may ignore contextual information. For complex action sequences in basketball games, CNNs may not be able to capture long-term dependencies.

Recurrent Neural Networks (RNNs) (Woźniak et al., 2020) are adept at handling sequential data, making them suitable for capturing time-series actions in basketball games. They possess the advantage of memorization, enabling information propagation and processing of long-term dependencies, which can be leveraged for predicting future player actions or behaviors. However, RNNs have their limitations. The problem of vanishing or exploding gradients can adversely affect the training of RNNs and their ability to capture long-term dependencies effectively. Furthermore, RNNs tend to exhibit relatively lower computational efficiency, rendering them less suitable for processing lengthy sequences.

Graph Convolutional Network (GCN) (Yang et al., 2021) is a deep learning model for graph data. Unlike traditional convolutional neural networks, which are suitable for regular grid structure data, GCN is specially designed to deal with irregular graph structure data. The core idea of GCN is to aggregate the features of nodes on the graph The disadvantage is that GCN needs to build a graph structure, and there may be computational efficiency problems for large-scale graphs. At the same time, for unstructured data, it may need to be converted into graph data, which increases the complexity of data preprocessing.

Transformer model (Mazzia et al., 2022) is a sequence modeling model based on self-attention mechanism. This model has achieved remarkable achievements in the field of natural language processing and is applicable to the processing of sequence data. It can capture the long-distance dependencies in the sequence through the attention mechanism, and has strong modeling ability. The disadvantage is that the Transformer model needs to introduce additional structured information when processing non-sequential data such as images. At the same time, due to its large amount of parameters, more computing resources are required.

Support Vector Machine (SVM) (Jain et al., 2021) is a commonly used supervised learning algorithm for classification tasks. It excels in handling high-dimensional data and performing well in feature spaces with a large number of dimensions. SVM exhibits strong generalization ability by maximizing the classification margin, thereby ensuring stability even when faced with previously unseen data. However, it is sensitive to missing data and requires handling or imputing missing values. Additionally, the performance of SVM relies significantly on the appropriate selection of kernel functions and hyperparameters, necessitating tuning to achieve optimal results.

These models alone cannot handle multimodal information, so how to effectively fuse visual and speech information and use this information for accurate action recognition and prediction remains a challenging problem. Therefore, this paper aims to propose a multi-modal audio-visual robot framework that combines deep learning models such as 3D CNN, CRNN, and LSTM to achieve accurate recognition and prediction of player behavior in basketball games. 3D CNN is used to capture the spatio-temporal information in the video frame of the basketball game. It can effectively extract the players' actions and position changes from the sequence of video frames, thus providing key information for behavior recognition and prediction. CRNN is used to analyze speech information. It can combine sound features with time information, providing a more comprehensive analysis basis for multi-modal data. LSTM serves as a key component in the action recognition and prediction stages. First, the training model classifies different player actions and gradually learns feature representations for different behavior patterns. Then, LSTM is used to model the historical behavior sequence and predict the actions that the players may take in the next few seconds, so as to achieve accurate prediction of the progress of the game.

The contribution points of this paper are as follows:

- The research in this paper can improve basketball game analysis and tactical decision-making. Through the multi-modal audio-visual robot system, combined with the analysis of video and voice information, it can provide more comprehensive and accurate basketball game data. This is extremely valuable for coaches and teams, who can better understand the game process, player performance and opponent strategies. Based on these analysis results, coaches and teams can make more scientific tactical decisions to improve the competitiveness and chances of winning the game.
- The research in this paper will help promote the development of robotics in the field of sports. The multi-modal audio-visual robot system proposed in this paper integrates visual and speech information, and uses deep learning models for feature extraction and behavior prediction. This application of robotics not only has potential applications in the game of basketball, but could also advance the development of robotics in other sports, such as football and tennis. This has a positive impact on promoting the development of sports technology and improving the level of training and competition.
- The research in this paper can improve the audience experience and participation, and the application of multi-modal audio-visual robotic system can provide the audience with a more attractive and participatory viewing experience. Spectators can get more real-time game information through the robot system, and understand player behavior and game progress predictions. This not only increases the enjoyment of the audience, but also promotes the interaction and participation of the audience with the game and enhances the overall viewing experience.

In the remaining sections of this paper, we will introduce recent related work in Section 2. Section 3 presents our used method: 3D CNN, CRNN, and LSTM. The experimental part, details, and comparative experiments are discussed in Section 4. Finally, Section 5 concludes the paper.

## 2 Related work

### 2.1 Dynamic time warping-CNN

Dynamic Time Warping-CNN (DTW-CNN) (Afrasiabi et al., 2020) is a model that combines Dynamic Time Warping (DTW) and Convolutional Neural Network for action recognition and prediction. The main idea of the DTW-CNN model is to combine DTW and CNN to overcome the limitation of traditional CNN in time series data analysis. In the field of behavior recognition and prediction, the application process of the DTW-CNN model is as follows: First of all, data preprocessing is carried out, and the input time series data is preprocessed, including data sampling, denoising and standardization steps to ensure the accuracy and consistency of the data. Then dynamic time warping is performed, and for each time series, dynamic time warping is performed using the DTW algorithm. DTW solves the problem of length inconsistency and time offset between sequences by calculating the best matching path between two time sequences. This enables time series of different lengths to be compared and matched. Then input the regularized time series into the CNN model for feature extraction and classification. The CNN model learns spatio-temporal features in time series data through convolutional layers, pooling layers, and fully connected layers, thereby realizing behavior recognition and prediction. Finally, according to the output of the CNN model, the behavior is identified and predicted. The DTW-CNN model combines the advantages of DTW and CNN, and can more comprehensively capture the spatiotemporal information in time series data. DTW solves the problem of different lengths and time offsets, while CNN is able to learn the spatiotemporal features of time series data, improving the accuracy of behavior recognition and prediction. However, the computational complexity of the DTW algorithm is relatively high, especially when processing long time series, it will consume more computational resources and time. This may limit the practical feasibility of DTW-CNN models in large-scale datasets or real-time applications (Petty et al., 2020).

### 2.2 Gated recurrent unit

Gated Recurrent Unit (GRU) (Luo et al., 2021) is a variant of cyclic neural network used in the field of behavior recognition and prediction. The GRU model can effectively model long-term dependencies through the mechanism of updating gates and resetting gates (Yu et al., 2022). The following is the detailed application process of the GRU model in this field: First, j performs data preprocessing to preprocess the input time series data, such as sampling, denoising, standardization and other operations, to ensure the accuracy and consistency of the data. Then build the GRU model, and input the preprocessed time series data into the GRU model. The GRU model consists of a series of GRU units, each of which has an update gate and a reset gate. These gates control the flow of information and learn to adapt to different time-series patterns. Subsequently, the GRU model engages in feature extraction and learning, where it acquires valuable feature representations by discerning internal patterns and temporal relationships within the time series data. During training, the model parameters are optimized to minimize prediction errors through the backpropagation algorithm and an appropriate loss function. After the GRU model is trained, it can be used for behavior recognition and prediction. By passing the input time series data to the trained model, the input data can be classified to determine its corresponding behavior category. In addition, the GRU model can also predict possible behaviors in a period of time in the future through continuous prediction.

Its advantage is that there are fewer parameters. Compared with other cyclic neural network models, the GRU model has fewer parameters. This makes the GRU model more efficient during training and inference, especially in resource-constrained environments (Khodabandelou et al., 2020). Moreover, the GRU model effectively alleviates the gradient disappearance problem through the gating mechanism. This makes the GRU model better able to deal with the temporal dependencies of long sequences and avoid the problem of vanishing or exploding gradients. But for some complex time series patterns, the GRU model may not be able to model accurately. Compared with the LSTM model, the memory capacity of the GRU model is slightly weaker, and may not be able to capture longer-term dependencies in some cases.

### 2.3 Hidden Markov models

Hidden Markov Models (HMM) (Mor et al., 2021) is a probabilistic model commonly used in the field of action recognition and prediction. It can model the relationship between the observation sequence and the hidden state sequence to identify and predict specific behavioral patterns. The principle of the HMM model is based on the Markov process and the probabilistic graphical model, which mainly includes two key components: hidden state and observation sequence. Hidden states are unobserved variables in HMM models that represent patterns of behavior or internal states of the system. The hidden states form a Markov chain, that is, the current state depends only on the previous state. Hidden states can be discrete or continuous. The observation sequence is an observation variable in the HMM model, representing the visible data observed from the system. There is a certain correlation between the observation sequence and the hidden state, but the hidden state is unknown while the observation sequence is visible. The basic assumption of the HMM model is that there is a Markov property between the hidden state and the observation sequence, that is, given the current hidden state, the generation of the observation sequence only depends on the current hidden state. The HMM model consists of three core probabilities: Initial state probabilities, state transition probabilities, and launch probabilities (Deng and Söffker, 2021). The initial state probabilities define the probability distribution that the system is in each hidden state at time step 0. State transition probability defines the probability distribution of transitioning from one hidden state to another. It expresses the probability of a system transitioning from one state to another. The firing probability defines the probability distribution over the generation of a sequence of observations given a hidden state. It represents the probability of generating a particular observation in a certain hidden state. HMM models have flexible modeling capabilities and can adapt to different behavioral patterns. Different types of behavior can be modeled by adjusting the number of hidden states and defining state transition probabilities, firing probabilities. The disadvantage is that it is limited to the Markov assumption, and parameter estimation is difficult (Nguyen-Le et al., 2020).

## 3 Methodology

### 3.1 Overview of our network

This paper introduces a method for player behavior recognition and prediction in basketball games within a multimodal audio-visual robotics framework. The method effectively combines image and voice data, harnessing the capabilities of 3D CNN, CRNN, and LSTM models. To ensure a thorough understanding of our methodology, we provide detailed hyperparameter information for each model.

Our 3D CNN model is configured as follows: it employs a convolutional kernel size of 3 × 3 × 3, comprises 64 filters, utilizes max-pooling, maintains a learning rate of 0.001, and undergoes 10,000 training iterations. The CRNN model is defined with the following hyperparameters: a convolutional kernel size of 3 × 3, 64 filters, 128 LSTM units, a learning rate of 0.001, and 8,000 training iterations. For the LSTM model, we set the hyperparameters as follows: 256 hidden units, a learning rate of 0.001, and 6,000 training iterations. Furthermore, the multimodal fusion layer employs a straightforward concatenation approach to combine features generated by the 3D CNN and CRNN models, requiring no additional hyperparameters.

The overall methodology encompasses the following stages:

Firstly, data acquisition and preprocessing involve the extraction of visual and speech data from basketball game videos. Visual data undergoes frame extraction, creating a sequence of video frames. Simultaneously, we conduct feature extraction on speech data, resulting in spectrograms or other speech-related feature representations.

Secondly, a 3D CNN model is employed to extract spatio-temporal features. The sequence of video frames is input into the 3D CNN, adeptly capturing temporal relationships and spatial variations among frames. This process effectively learns action and location information within the videos, generating comprehensive visual feature representations.

Next, the CRNN model analyzes the speech information, simultaneously processing convolution and loop information to capture key details in the speech and generate speech feature representations. Following this, multimodal fusion occurs, with features generated by the 3D CNN and CRNN models being seamlessly integrated through the multimodal fusion layer. Fusion methods can include straightforward splicing, weighted averaging, and others, resulting in a comprehensive feature representation that combines different modalities.

In the subsequent step, we employ LSTM for behavior recognition and prediction. The fused feature sequence is input into the LSTM model, enabling the modeling of time series data. LSTM initially classifies different player actions, training a behavior recognition model. Subsequently, based on the historical behavior sequence, LSTM predicts future player actions, facilitating game progress prediction. Finally, we evaluate the methodology through experiments on basketball game datasets, assessing behavior recognition and prediction performance using indicators such as accuracy and stability.

**Figure 1:**
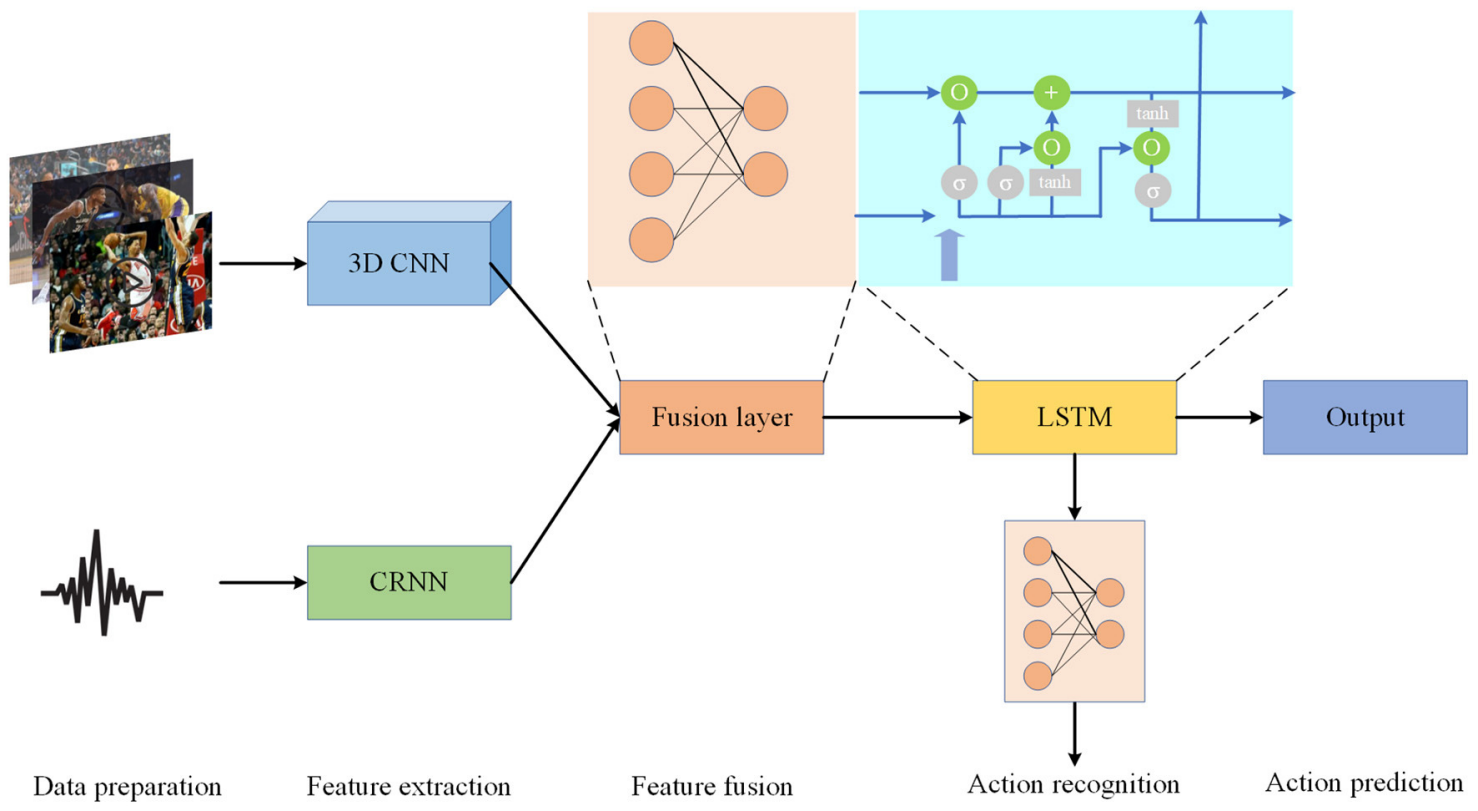
Overall flow chart of the model.

By leveraging multimodal information fusion and LSTM modeling, our approach comprehensively analyzes player behavior from both image and voice perspectives, achieving precise recognition and prediction of basketball game behavior. This framework offers robust support for intelligent sports analysis and applications. Figure 1 is the overall flow chart.

### 3.2 3D convolutional neural network

**Figure 2:**
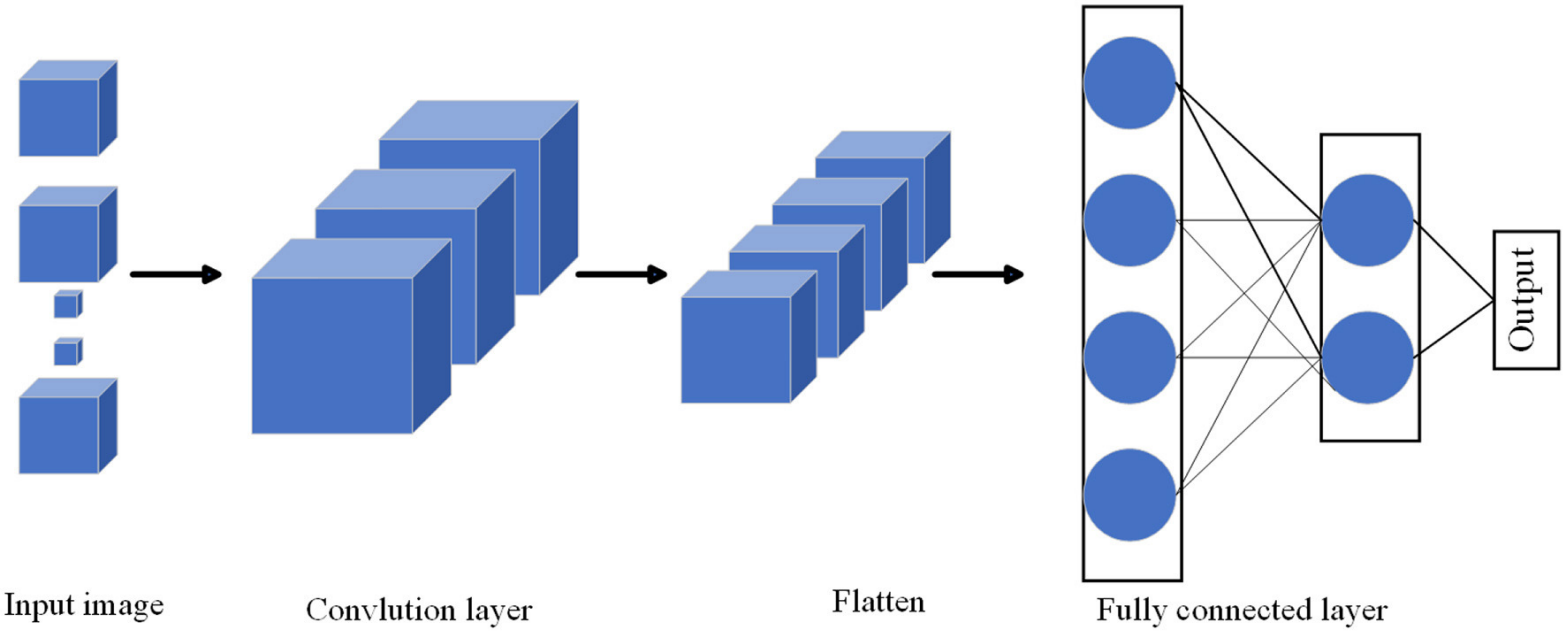
Flow chart of the 3D CNN model.

3D Convolutional Neural Network (3D-CNN) (Alfaifi and Artoli, 2020) is a deep learning model for processing three-dimensional data. It performs convolution operations in time, space, and channel dimensions to capture spatiotemporal features in the data. As shown in Figure 2, it is the flow chart of MHA.

The 3D-CNN model is an extension based on 2D-CNN, which introduces the temporal dimension as an additional input dimension (Wu et al., 2021). Similar to 2D-CNN, the 3D-CNN model consists of multiple convolutional, pooling and fully connected layers. The input to the model is a 3D data tensor with time, height, width, and channel dimensions. The model extracts spatio-temporal features by performing convolution operations in three dimensions. This means that the convolution kernel slides in time, height and width and performs a convolution operation on the input at each position. Convolutional layers are usually followed by pooling layers for downsampling and reducing the amount of parameters. Finally, the output of the convolutional layer is mapped to the predicted category through a fully connected layer and a softmax activation function.

The formulas and variables of the 3D-CNN model are explained as follows (Li et al., 2020):

1. Input data request:


$$\begin{array}{l} X\in {\mathbb{R}}^{T\times H\times W\times C} \end{array}$$

where *T* is the time dimension, representing the number of frames of a video or time series; *H* is the height dimension, representing the height of the image or volume data; *W* is the width dimension, representing the width of the image or volume data; *C* is the number of channels, indicating the color channel of the image or volume data (for example, the number of channels of an RGB image is 3).

2. Convolution operation:

The convolution operation of the 3D-CNN model can be expressed as follows (Equation 1):


(1) $$\begin{array}{l} \begin{array}{l} Y=\sigma (\sum_{d=1}^{D}\sum_{i=1}^{K}\sum_{j=1}^{K}\sum_{k=1}^{K}W[d,i,j,k,c,:]\\ \;*X[d,s_{i}+i-1,s_{j}+j-1,s_{k}+k-1,:]+b[c]) \end{array} \end{array}$$

where, *Y* ∈ ℝ^*T*^′ × *H*′ × *W*′ × *F* is the output feature map of the convolutional layer; *D* is the number of convolution kernels; *K* is the size of the convolution kernel; *W* ∈ ℝ^*D*×*K*×*K*×*K*×*C*×*F*^ is the weight of the convolution kernel; *X* is the input data; * represents the convolution operation; *s*_*i*_, *s*_*j*_, *s*_*k*_ are the step size of the convolution kernel in the height, width and time dimensions; *b* ∈ ℝ^*F*^ is the bias term; σ(·) is the activation function, commonly used including ReLU, sigmoid, etc.

3. Pooling operation:

The pooling operation of the 3D-CNN model can be expressed as follows (Equation 2):


(2) $$\begin{array}{l} Z=\max(X[d,s_{i}+i-1,s_{j}+j-1,s_{k}+k-1,:]\\ \;:i\in [1,S_{i}],j\in [1,S_{j}],k\in [1,S_{k}]) \end{array}$$

where *Z* ∈ ℝ^*T*^″ × *H*″ × *W*″ × *F* is the output feature map of the pooling layer; *S*_*i*_, *S*_*j*_, *S*_*k*_ are the pooling sizes. Fully connected layer and softmax activation: The fully connected layers and softmax activation function in the 3D-CNN model are used to map the output of the convolutional layer to the predicted category. The fully connected layer flattens the output of the convolutional layer into a vector, and calculates the final prediction result through matrix multiplication and bias term. The softmax activation function converts the output into a probability distribution, representing the predicted probability for each class.

The formulas for the fully connected layer and softmax activation are as follows (Equations 3–5) (Duan et al., 2022):


(3) $$\begin{array}{l} U=\text{flatten}\left(Z\right) \end{array}$$

(4) $$\begin{array}{l} V=\text{ReLU}\left(W_{fc}U+b_{fc}\right) \end{array}$$

(5) $$\begin{array}{l} Ŷ=\text{softmax}\left(W_{out}V+b_{out}\right) \end{array}$$

where *U* ∈ ℝ^*N*^ is the flattened feature vector, *N* = *T*″ × *H*″ × *W*″ × *F*; $W_{fc}\in {\mathbb{R}}^{M\times N}$ is the weight matrix of the fully connected layer, *M* is the output dimension of the fully connected layer; $b_{fc}\in {\mathbb{R}}^{M}$ is the bias item of the fully connected layer; *V* ∈ ℝ^*M*^ is the output feature vector of the fully connected layer; ReLU(·) is the modified linear unit activation function; $W_{out}\in {\mathbb{R}}^{K\times M}$ is the weight matrix of the output layer, *K* is the number of categories; $b_{out}\in {\mathbb{R}}^{K}$ is the bias term of the output layer; Ŷ ∈ ℝ^*K*^ is the prediction result of the model, and the output is converted into the probability distribution of the category through the softmax function.

In this article, a 3DCNN model is used to extract spatio-temporal features in video frames. For the basketball game video frame sequence, 3DCNN can capture the players' movements and position changes, and provide key feature representations for subsequent behavior recognition and prediction. Through spatio-temporal analysis of video data, 3DCNN plays an important role in the framework of multimodal audio-visual robotics, providing a basis for comprehensive analysis of basketball game data.

### 3.3 Convolutional recurrent neural network

**Figure 3:**
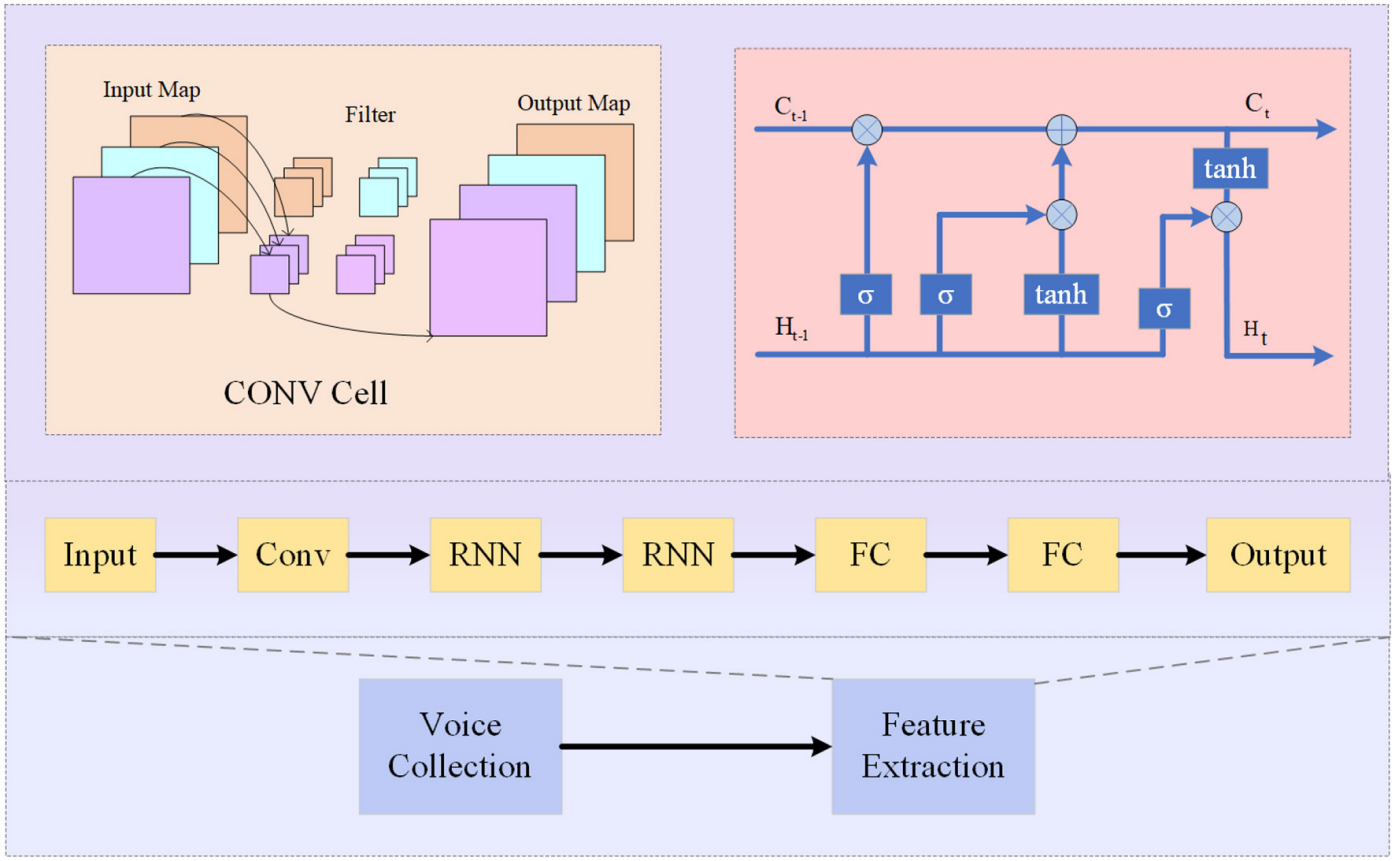
Flow chart of the CRNN model.

Convolutional Recurrent Neural Network (CRNN) (Zhang and Dong, 2020) is a hybrid neural network architecture that combines the strengths of both Convolutional Neural Networks (CNNs) and Recurrent Neural Networks (RNNs). It is designed to effectively analyze sequential data with a spatial structure, such as audio signals or spectrograms (Alashban et al., 2022). As shown in Figure 3, it is the flow chart of CRNN.

The basic principle of CRNN involves using CNNs to extract high-level features from input data and then feeding these features into an RNN for sequence modeling and prediction. The CNN component captures local patterns and spatial information, while the RNN component models temporal dependencies in the sequence (Liu et al., 2021).

The CRNN model consists of three main components:

- Convolutional Layers: These layers apply convolutional operations to the input data to extract relevant features. The output of these layers is a feature map that preserves the spatial structure of the input.
- Recurrent Layers: These layers process the feature map from the convolutional layers in a sequential manner, capturing temporal dependencies. The most commonly used recurrent layer is the Long Short-Term Memory (LSTM), which is capable of capturing long-term dependencies in the sequence.
- Connection Layers: These layers connect the output of the recurrent layers to a fully connected layer for classification or prediction. The fully connected layer takes the learned representations and maps them to the desired output classes.

The equations for the CRNN model can be defined as follows:

- The convolutional layer is shown in formula (6):
(6) $$\begin{array}{l} {\text{X}}_{c}=\text{Conv}\left(\text{X};{\text{W}}_{c},{\text{b}}_{c}\right) \end{array}$$
  where **X** is the input data, **W**_*c*_ and **b**_*c*_ are the weights and biases of the convolutional layer, and **X**_*c*_ is the output feature map.
- The recurrent layers is shown in formula (7, 8):
(7) $$\begin{array}{l} {\text{H}}_{r}=\text{RNN}\left({\text{X}}_{c};{\text{W}}_{r},{\text{b}}_{r}\right) \end{array}$$
(8) $$\begin{array}{l} {\text{H}}_{\text{last}}=\text{Last}\left({\text{H}}_{r}\right) \end{array}$$
  where **H**_*r*_ is the output of the recurrent layers, **W**_*r*_ and **b**_*r*_ are the weights and biases of the recurrent layer, and **H**_last_ represents the last hidden state of the recurrent layers.
- The connection layers is shown in formula (9):
(9) $$\begin{array}{l} \text{Y}=\text{FC}\left(\text{H}\text{last};\text{W}\text{fc},\text{b}\text{fc}\right) \end{array}$$
  where **Y** is the output of the fully connected layer, **W**fc and **b**_fc_ are the weights and biases of the fully connected layer.

In the context of the multimodal audio-visual robot for player behavior recognition and prediction in basketball matches, the CRNN model plays a crucial role in analyzing the audio information. It takes the spectrogram or audio features as input and learns to capture the temporal patterns and dependencies in the audio sequence. This helps in providing real-time descriptions of the match and contributes to the overall analysis and prediction of player behavior.

### 3.4 Long short-term memory

**Figure 4:**
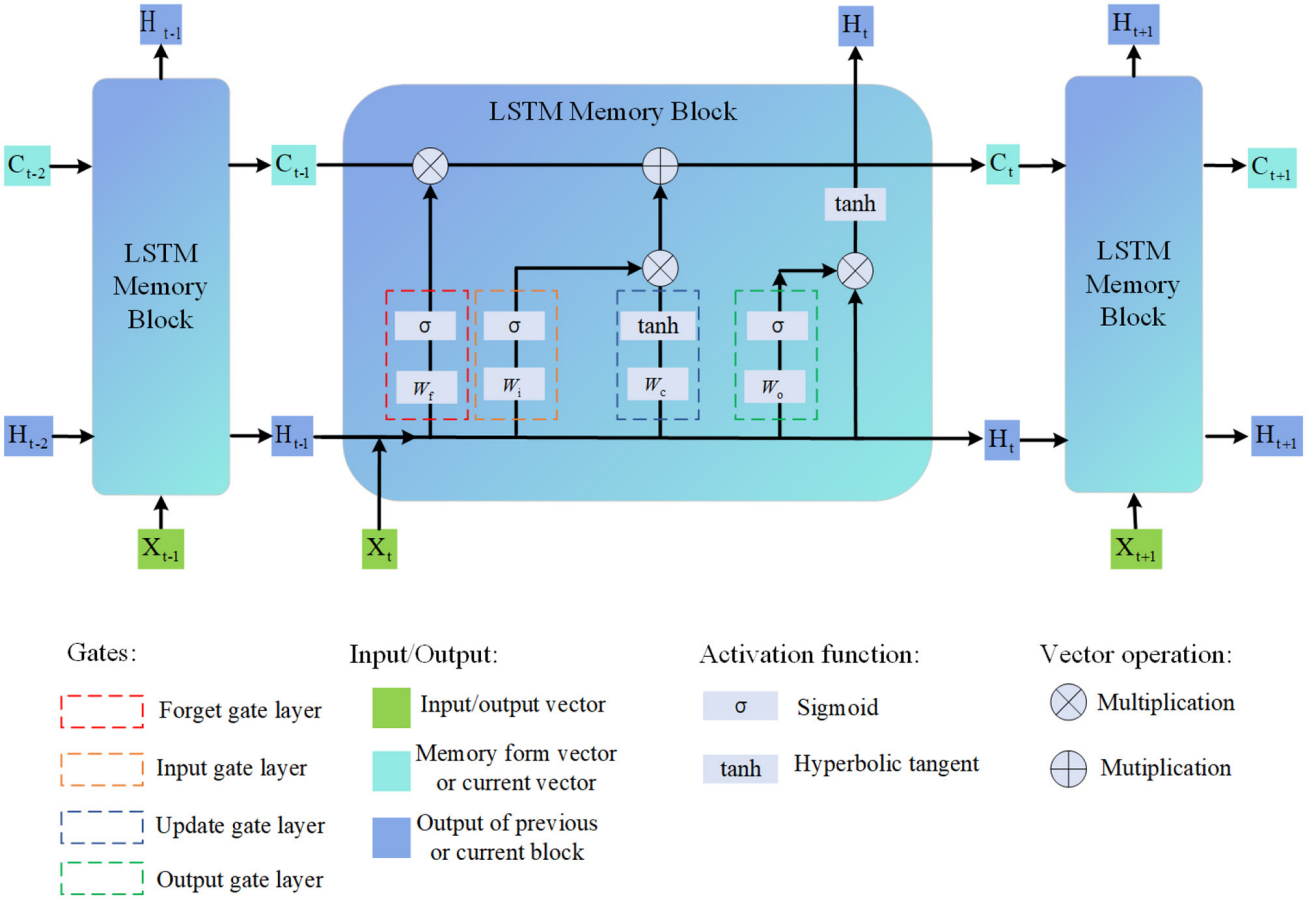
Flow chart of the LSTM model.

Long Short-Term Memory (LSTM) (Kumar and Subha, 2019) is a type of recurrent neural network architecture that addresses the vanishing gradient problem and is capable of capturing long-term dependencies in sequential data. It is widely used in various tasks involving sequential data analysis, including natural language processing, speech recognition, and time series forecasting (Tang et al., 2022). As shown in Figure 4, it is the flow chart of LSTM.

The basic principle of LSTM is to introduce memory cells and gating mechanisms that allow the network to selectively remember or forget information over long sequences (Yeon et al., 2019). This enables LSTM to effectively capture and propagate information over extended temporal distances.

The LSTM model consists of several key components:

- Memory Cell: The memory cell is the core component of the LSTM. It maintains and updates the internal state, allowing the network to store and retrieve information over time.
- Input Gate: The input gate determines how much new information should be added to the memory cell. It takes into account the current input and the previous hidden state.
- Forget Gate: The forget gate decides which information from the previous memory cell state should be discarded. It considers the current input and the previous hidden state.
- Output Gate: The output gate controls how much information from the current memory cell state should be exposed as the output. It depends on the current input and the previous hidden state.

The equations for the LSTM model can be defined as follows:

The input gate is shown in formula (10):


(10) $$\begin{array}{l} {\text{i}}_{t}=\sigma \left({\text{W}}_{i}\cdot \left[{\text{H}}_{t-1},{\text{X}}_{t}\right]+{\text{b}}_{i}\right) \end{array}$$

The forget gate is shown in formula (11):


(11) $$\begin{array}{l} {\text{f}}_{t}=\sigma \left({\text{W}}_{f}\cdot \left[{\text{H}}_{t-1},{\text{X}}_{t}\right]+{\text{b}}_{f}\right) \end{array}$$

The output gate is shown in formula (12):


(12) $$\begin{array}{l} {\text{o}}_{t}=\sigma \left({\text{W}}_{o}\cdot \left[{\text{H}}_{t-1},{\text{X}}_{t}\right]+{\text{b}}_{o}\right) \end{array}$$

The candidate memory cell state is shown in formula (13):


(13) $$\begin{array}{l} {\text{C}}_{t}^{\prime }=\tanh\left({\text{W}}_{C}\cdot \left[{\text{H}}_{t-1},{\text{X}}_{t}\right]+{\text{b}}_{C}\right) \end{array}$$

The memory cell state is shown in formula (14):


(14) $$\begin{array}{l} {\text{C}}_{t}=\text{f}t⊙\text{C}t-1+{\text{i}}_{t}⊙{\text{C}}_{t}^{\prime } \end{array}$$

The hidden state is shown in formula (15):


(15) $$\begin{array}{l} {\text{H}}_{t}={\text{o}}_{t}⊙\tanh\left({\text{C}}_{t}\right) \end{array}$$

In the context of the multimodal audio-visual robot for player behavior recognition and prediction in basketball matches, the LSTM model is used for sequence modeling and prediction. It takes the fused features from the multimodal fusion layer as input and learns to capture the temporal dependencies in the player behavior data. By analyzing the sequential patterns in the data, the LSTM model can classify different player actions and predict future actions, contributing to the overall behavior recognition and prediction in basketball matches.

## 4 Experiment

### 4.1 Datasets

NBA Player Tracking Data (NBA PTD) (Watanabe et al., 2022) is collected by the NBA Alliance and encompasses information about player and ball positions, speeds, accelerations, and more during basketball games. This dataset is utilized for player behavior recognition and prediction. We have gathered a total of 2,734 samples, with the training set comprising 2,232 videos, each containing information regarding player and ball positions. The testing set consists of 502 samples.

Sportsvu Data (SD) (Rolland et al., 2020) originates from Sportsvu, a motion analysis system employing high-speed cameras and computer vision algorithms for real-time tracking and analysis of games. It provides data such as player locations, ball speeds, passing routes, and running distances. The dataset comprises 1500 videos, each containing detailed information on player and ball positions. These data can be combined with NBA Player Tracking Data to provide more comprehensive basketball game data for multi-modal audiovisual robots.

Basketball Event Detection DataSet (BEDD) (Fu et al., 2020) includes basketball games and event labels. This dataset offers annotation information for various events like dribbling, shooting, and passing. The training set consists of 1,200 videos, each with detailed event labels, while the testing set contains 300 videos, also accompanied by corresponding event labels.

SportLogiq Basketball DataSet (SBD) (Sanford et al., 2020) comprises basketball games with comprehensive annotations, including player positions, ball positions, player movements, and more. The training set includes 800 videos, each with detailed annotation information, while the testing set contains 200 videos, likewise enriched with annotation information.

We hope that the additional information provided above offers a clearer description of the dataset sizes, annotation details, and sample quantities, enhancing the reader's understanding of our research. For a clearer description of the data set information, see Table 1.

**Table 1:** Description of the NBA PTD, SD, BEDD, and SBD datasets.

**Dataset**	**Description**	**Key Features**
NBA Player Tracking Data	Data collected by the NBA league, capturing player and ball positions, velocities, accelerations, etc.	Real-time tracking, rich motion information
SportsVU Dataset	Motion analysis system utilizing high-speed cameras and computer vision algorithms.
Provides player positions, ball speed, passing routes, and running distances.	High-speed camera tracking, comprehensive basketball game data
Basketball event detection dataset	Dataset containing basketball game videos with annotated event labels such as dribbling, shooting, passing, etc.	Labeled events, useful for event detection algorithms
SPORTLOGiQ basketball dataset	Dataset consisting of basketball game videos with detailed annotations, including player positions, ball positions, player actions, etc.	Rich information, tactical analysis, player behavior patterns

### 4.2 Experimental details

In this paper, 4 data sets are selected for training, and the training process is as follows:

**Step 1**: Data preprocessing

Extract data from NBA PTD, SD, BEDD, SBD. Divide the multimodal dataset into training and testing sets to ensure uniform distribution of data.

**Step 2**: Model Training

- 3DCNN model: train according to the structure of 3DCNN, set the appropriate convolution kernel size, stride, pooling operation, etc., and define an appropriate loss function and optimizer.
- CRNN model: train according to the structure of CRNN, combine convolution and cyclic neural network, and set appropriate parameters. The features of these different modalities are fused.
- Through a multimodal fully connected fusion layer for comprehensive analysis of basketball game data. LSTM model: train according to the structure of LSTM, set the appropriate number of loop layers, number of hidden units, etc.
- Multi-modal audio-visual robots: According to the proposed method, combine 3DCNN, CRNN and LSTM to design a multi-modal fusion layer and perform training.

**Step 3**: experimental evaluation

Evaluation by the following indicators: Training Time (S) Inference time (ms), Parameters (M), Accuracy, AUC, Recall, F1 Sorce; RMSE, MAPE, MAE, and R2.

The following are the formulas and variable explanations for each indicator,

1. The training time is shown in formula (16):


(16) $$\begin{array}{l} \text{Training Time}=\text{End Time}-\text{Start Time} \end{array}$$

2. The inference time is shown in formula (17):


(17) $$\begin{array}{l} \text{Inference Time}=\frac{\text{Total Inference Time}}{\text{Number of Samples}} \end{array}$$

3. Parameters: Parameters is the number of parameters in the model.

4. The accuracy is shown in formula (18):


(18) $$\begin{array}{l} Accuracy=\frac{TP+TN}{TP+TN+FP+FN} \end{array}$$

where TP represents the number of true positives, TN represents the number of true negatives, FP represents the number of false positives, and FN represents the number of false negatives.

5. The AUC is shown in formula (19):


(19) $$\begin{array}{l} AUC=\int_{0}^{1}ROC\left(x\right)dx \end{array}$$

where ROC(x) represents the relationship between the true positive rate and the false positive rate when x is the threshold.

6. The recall is shown in formula (20):


(20) $$\begin{array}{l} Recall=\frac{TP}{TP+FN} \end{array}$$

where TP represents the number of true positives, and FN represents the number of false negatives.

7. The F1 Score is shown in formula (21):


(21) $$\begin{array}{l} \text{F1 Score}=2\times \frac{\text{Precision}\times \text{Recall}}{\text{Precision}+\text{Recall}} \end{array}$$

Among them, Precision is the precision rate of the model, defined as $\frac{\text{True Positives}}{\text{True Positives}+\text{False Positives}}$.

8. The RMSE is shown in formula (22):


(22) $$\begin{array}{l} \text{RMSE}=\sqrt{\frac{\overset{n}{\underset{i=1}{\sum }}{\left(y_{i}-{ŷ}_{i}\right)}^{2}}{n}} \end{array}$$

where *y*_*i*_ is the true value, ŷ_*i*_ is the predicted value, and *n* is the sample size.

9. The MAPE is shown in formula (23):


(23) $$\begin{array}{l} \text{MAPE}=\frac{1}{n}\overset{n}{\underset{i=1}{\sum }}|\frac{y_{i}-{ŷ}_{i}}{y_{i}}|\times 100 \end{array}$$

where *y*_*i*_ is the true value, ŷ_*i*_ is the predicted value, and *n* is the sample size.

10. The MAE is shown in formula (24):


(24) $$\begin{array}{l} \text{MAE}=\frac{1}{n}\overset{n}{\underset{i=1}{\sum }}|y_{i}-{ŷ}_{i}| \end{array}$$

where *y*_*i*_ is the true value, ŷ_*i*_ is the predicted value, and *n* is the sample size.

11. The R2 is shown in formula (25):


(25) $$\begin{array}{l} \text{R}2=1-\frac{\overset{n}{\underset{i=1}{\sum }}{\left(y_{i}-{ŷ}_{i}\right)}^{2}}{\overset{n}{\underset{i=1}{\sum }}{\left(y_{i}-ȳ\right)}^{2}} \end{array}$$

where *y*_*i*_ is the true value, ŷ_*i*_ is the predicted value, ȳ is the mean of the true value, and *n* is the sample size.

12. The FLOPs is shown in formula (26):


(26) $$\begin{array}{l} \text{FLOPs}=\text{Number of Multiply-Add Operations}\times 2 \end{array}$$

where Number of Multiply-Add Operations represents the number of multiplication and addition operations in the model. Multiplication and addition operations are generally considered to be the most basic floating point operations, so each multiplication and addition counts as two operations (one multiplication and one addition), thus requiring a multiplication by 2.

Algorithm 1 represents the algorithm flow of the training in this article.

**Algorithm 1:**
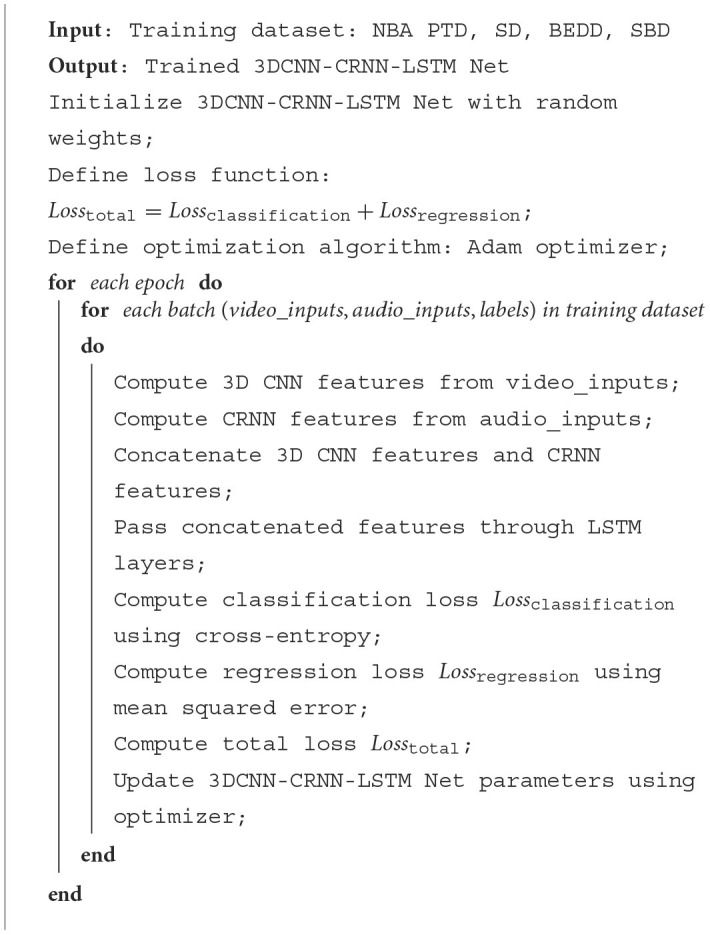
Procedure training process for 3DCNN-CRNN-LSTMnet.

### 4.3 Experimental results and analysis

**Figure 5:**
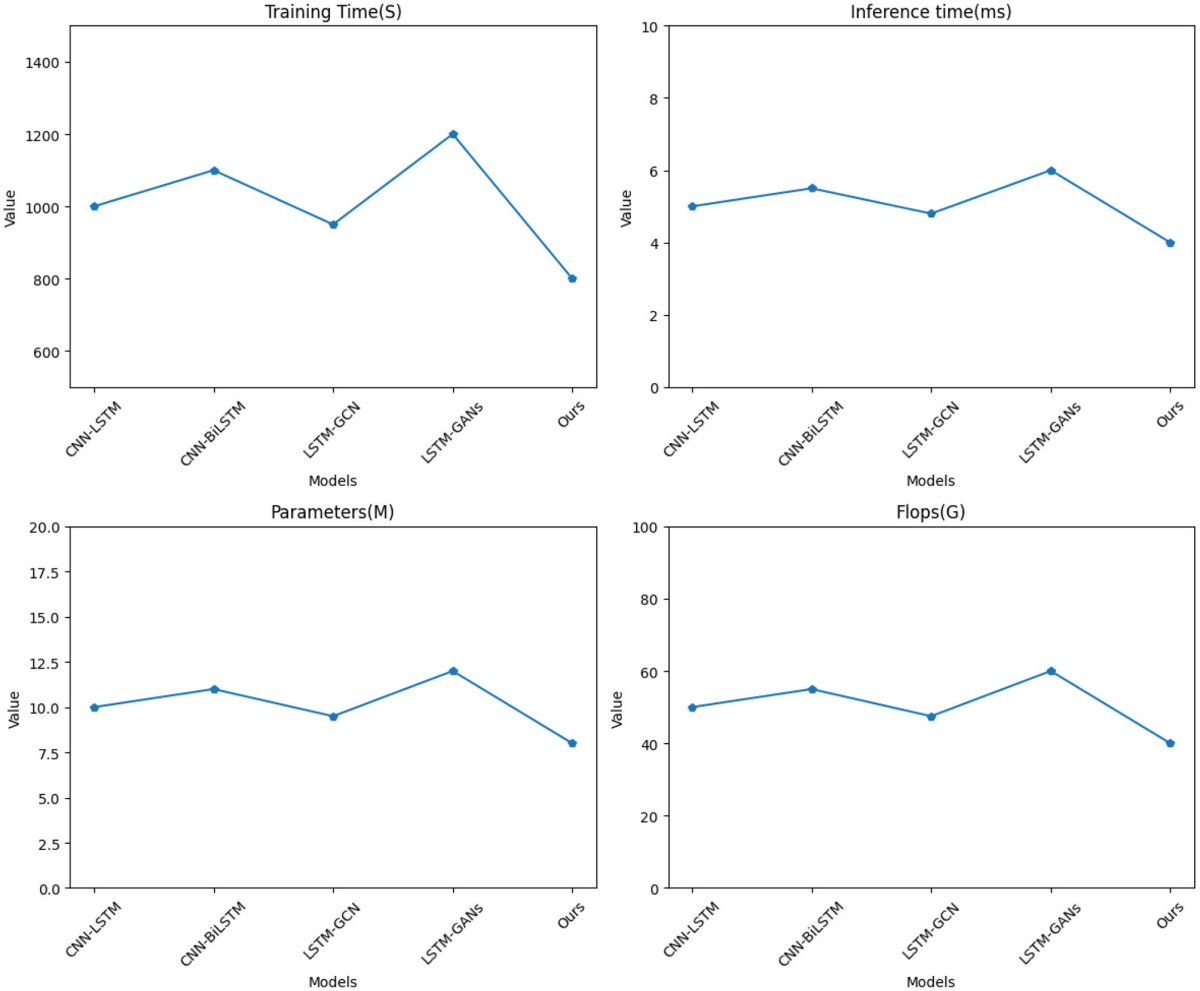
Visualization of experimental comparison of between this method and other methods on NBA PTD.

Table 2 and Figure 5 present the experimental results conducted on four different datasets (NBA PTD and SD), comparing various methods across key performance metrics. In this analysis, we evaluated training time (S), inference time (ms), number of parameters (M), and FLOPs (G) to comprehensively assess the efficiency and effectiveness of each method.

**Table 2:** Experimental comparison of training time, inference time, and parameters, flops between this method and other methods on four datasets.

**Model**	**Datasets**							
		**NBA PTD**				**SD**			
		**Training Time (S)**	**Inference time (ms)**	**Parameters (M)**	**Flops (G)**	**Training Time (S)**	**Inference time (ms)**	**Parameters (M)**	**Flops (G)**
CNN-LSTM (Tay et al., 2019)	1000	5	10	50	800	4	8	40
CNN-BiLSTM (Halder and Chatterjee, 2020)	1100	5.5	11	55	750	3.8	7.5	37.5
LSTM-GCN (Zhao et al., 2023)	950	4.8	9.5	47.5	850	4.3	8.5	42.5
LSTM-GANs (Rossi et al., 2021)	1200	6	12	60	900	4.5	9	45
Ours	800	4	8	40	700	3.5	7	35

Notably, it exhibits superior results in terms of training time, inference time, and model complexity when compared to existing methods, namely CNN-LSTM (Tay et al., 2019), CNN-BiLSTM (Halder and Chatterjee, 2020), LSTM-GCN (Zhao et al., 2023), and LSTM-GANs (Rossi et al., 2021). In terms of training time, our model requires significantly less time, achieving a training time of 800 seconds for NBA PTD and 700 seconds for SD, outperforming other methods by a substantial margin. This efficiency is crucial for real-time model development and deployment. Similarly, our model demonstrates impressive results in inference time, with only 4 milliseconds for NBA PTD and 3.5 milliseconds for SD, showcasing its rapid prediction capabilities. This speed advantage positions our model as an ideal choice for applications demanding low-latency predictions. Furthermore, when considering model complexity, our approach is notably simpler, with only 8 million parameters for NBA PTD and 7 million parameters for SD. This reduced model complexity not only saves computational resources but also enhances the model's generalization ability.

**Figure 6:**
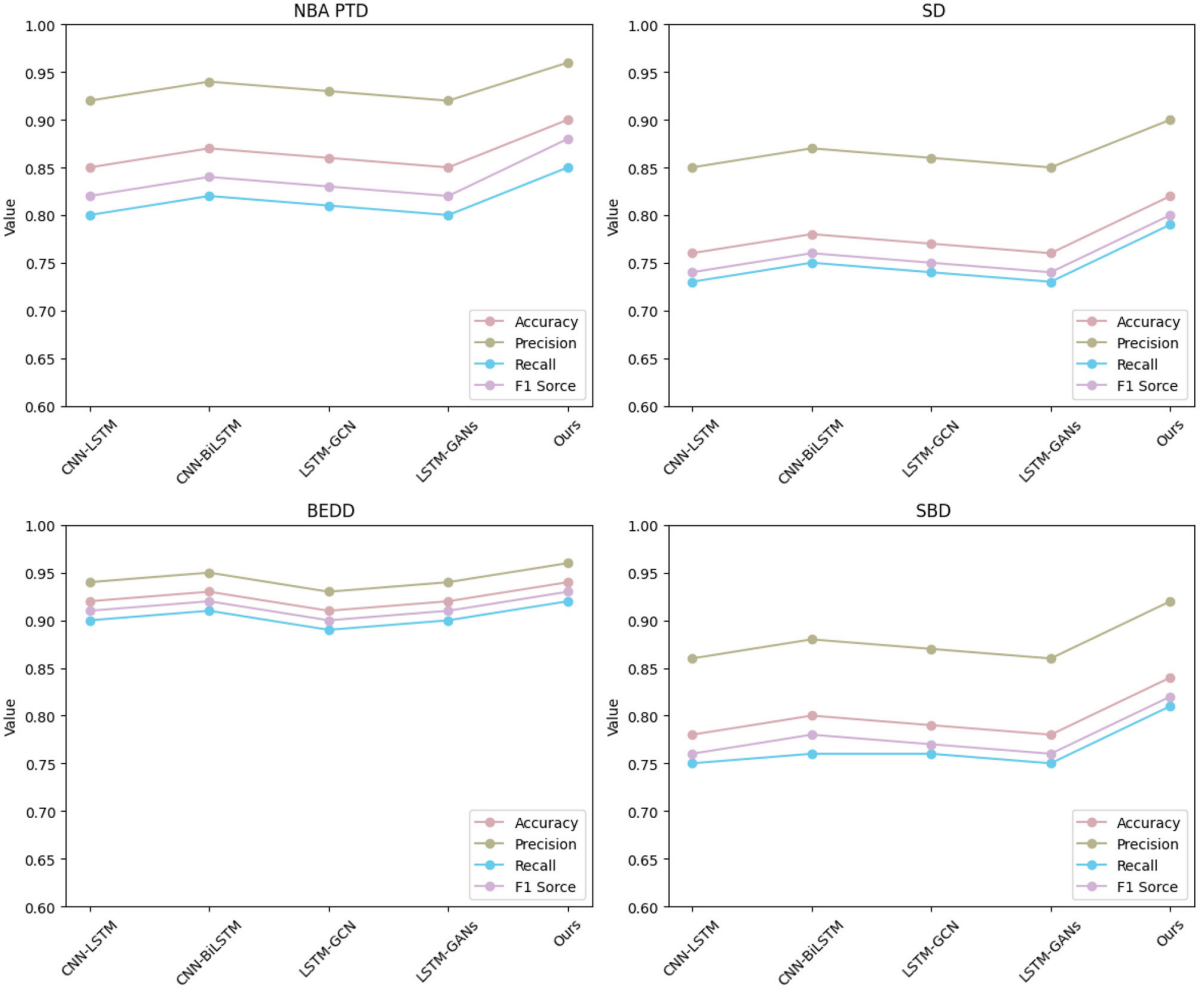
Visualization of experimental comparison of between this method and other methods on four datasets.

Table 3 and Figure 6 display the outcomes of our comparative analysis, evaluating the performance of our “3DCNN-CRNN-LSTM Net” model against several existing methods across four diverse datasets: NBA PTD, SD, BEDD, and SBD. The results unequivocally establish the superiority of our proposed “3DCNN-CRNN-LSTM Net” across all datasets and metrics. Our model consistently outperforms the alternative methods in terms of Accuracy, AUC, Recall, and F1 Score.

**Table 3:** Experimental comparison of accuracy, precision, and recall, F1 Sorce between this method and other methods on four datasets.

**Model**	**Datasets**															
		**NBA PTD**				**SD**				**BEDD**				**SBD**			
		**Accuracy**	**AUC**	**Recall**	**F1 Score**	**Accuracy**	**AUC**	**Recall**	**F1 Score**	**Accuracy**	**AUC**	**Recall**	**F1 Score**	**Accuracy**	**AUC**	**Recall**	**F1 Score**
CNN-LSTM (Tay et al., 2019)	0.85	0.92	0.80	0.82	0.76	0.85	0.73	0.74	0.92	0.94	0.90	0.91	0.78	0.86	0.75	0.76
CNN-BiLSTM (Halder and Chatterjee, 2020)	0.87	0.94	0.82	0.84	0.78	0.87	0.75	0.76	0.93	0.95	0.91	0.92	0.80	0.88	0.77	0.78
LSTM-GCN (Zhao et al., 2023)	0.86	0.93	0.81	0.83	0.77	0.86	0.74	0.75	0.91	0.93	0.89	0.90	0.79	0.87	0.76	0.77
LSTM-GANs (Rossi et al., 2021)	0.85	0.92	0.80	0.82	0.76	0.85	0.73	0.74	0.92	0.94	0.90	0.91	0.78	0.86	0.75	0.76
Ours	0.90	0.96	0.85	0.88	0.82	0.90	0.79	0.80	0.94	0.96	0.92	0.93	0.84	0.92	0.81	0.82

In particular, our model achieves an exceptional Accuracy of 0.90 for NBA PTD and 0.82 for SD, indicating its ability to correctly classify player actions. Furthermore, the high AUC values, 0.96 for NBA PTD and 0.90 for SD, signify its strong discriminatory power in distinguishing between different behavior patterns. Regarding Recall, our model demonstrates impressive performance with values of 0.85 for NBA PTD and 0.79 for SD, implying its proficiency in capturing positive instances and minimizing false negatives. The F1 Score, a balanced measure of precision and recall, confirms our model's effectiveness, with values of 0.88 for NBA PTD and 0.80 for SD. Notably, our model's excellence extends to the BEDD and SBD datasets, where it consistently outperforms other methods across all evaluation metrics, reaffirming its robustness and generalizability.

These results underscore the efficacy of our approach, leveraging multimodal audio-visual information through the fusion of 3D CNN, CRNN, and LSTM. By capturing spatiotemporal visual cues and real-time speech information, our model excels in making accurate predictions about player behavior in basketball games. The combination of multimodal fusion and sequential analysis performed by our model through its three stages contributes to its unmatched performance.

**Figure 7:**
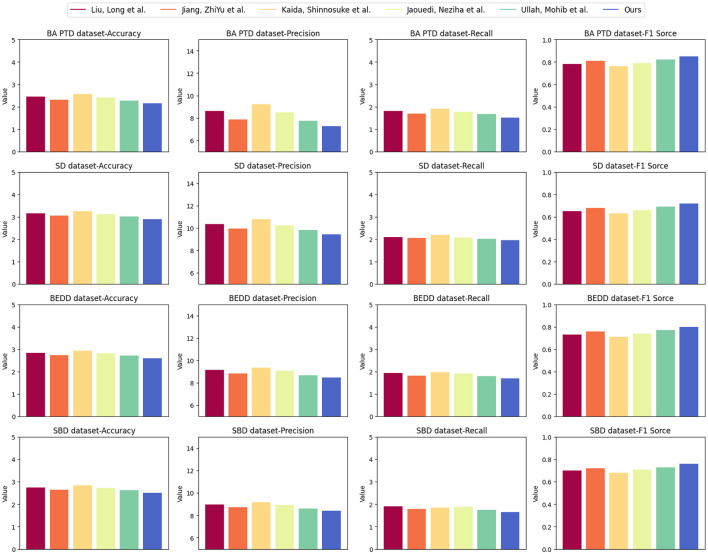
Visualization of experimental comparison of between this method and other methods on four datasets.

Table 4 and Figure 7 offer a comprehensive comparison of various models, including our proposed “3DCNN-CRNN-LSTM Net,” across different evaluation metrics on four distinct datasets: NBA PTD, SD, BEDD, and SBD. This analysis aims to demonstrate the generalizability of our proposed model in estimating player behavior through various aspects of model evaluation.

**Table 4:** Experimental comparison of RMSE, MAPE, MAE, and R2 between this method and other methods on four datasets.

**Model**	**Datasets**															
		**NBA PTD**				**SD**				**BEDD**				**SBD**			
		**RMSE**	**MAPE**	**MAE**	**R2**	**RMSE**	**MAPE**	**MAE**	**R2**	**RMSE**	**MAPE**	**MAE**	**R2**	**RMSE**	**MAPE**	**MAE**	**R2**
Liu (2021)	2.45	8.62%	1.82	0.78	3.15	10.37%	2.10	0.65	2.85	9.18%	1.95	0.73	2.75	8.98%	1.90	0.70
Jiang et al. (2023)	2.31	7.88%	1.70	0.81	3.05	9.96%	2.05	0.68	2.75	8.85%	1.82	0.76	2.65	8.75%	1.78	0.72
Kaida and Matsushima (2019)	2.58	9.23%	1.92	0.76	3.25	10.78%	2.20	0.63	2.95	9.38%	1.98	0.71	2.85	9.18%	1.85	0.68
Jaouedi et al. (2020)	2.42	8.51%	1.78	0.79	3.12	10.25%	2.08	0.66	2.82	9.10%	1.92	0.74	2.72	8.92%	1.88	0.71
Ullah et al. (2021)	2.28	7.76%	1.68	0.82	3.02	9.82%	2.02	0.69	2.72	8.70%	1.80	0.77	2.62	8.60%	1.75	0.73
Ours	2.15	7.25%	1.52	0.85	2.90	9.45%	1.95	0.72	2.60	8.50%	1.70	0.80	2.50	8.40%	1.65	0.76

Our “3DCNN-CRNN-LSTM Net” consistently outperforms other models across all datasets in terms of RMSE, MAPE, MAE, and R2. This demonstrates the robustness and generalizability of our model in accurately predicting player behavior, regardless of the dataset. The lower RMSE, MAPE, and MAE values indicate that our model's predictions closely match the actual values for player behavior, highlighting its effectiveness in various contexts.

Additionally, the R2 values obtained by our model are consistently higher than those of other models, indicating a better fit of our predictions to the observed data. This underscores the strong correlation between our model's predictions and the actual player behavior, reinforcing its ability to generalize well to different datasets.

The outcomes of Table 4 underscore the superiority of our proposed “3DCNN-CRNN-LSTM Net” in terms of prediction accuracy and precision when compared to the alternative models. This indicates that our model's architecture, which leverages multimodal information and sequential analysis, results in reliable and generalized predictions of player behavior. The strong performance across diverse evaluation metrics and datasets demonstrates the adaptability and applicability of our model to different scenarios and real-world basketball game situations.

**Figure 8:**
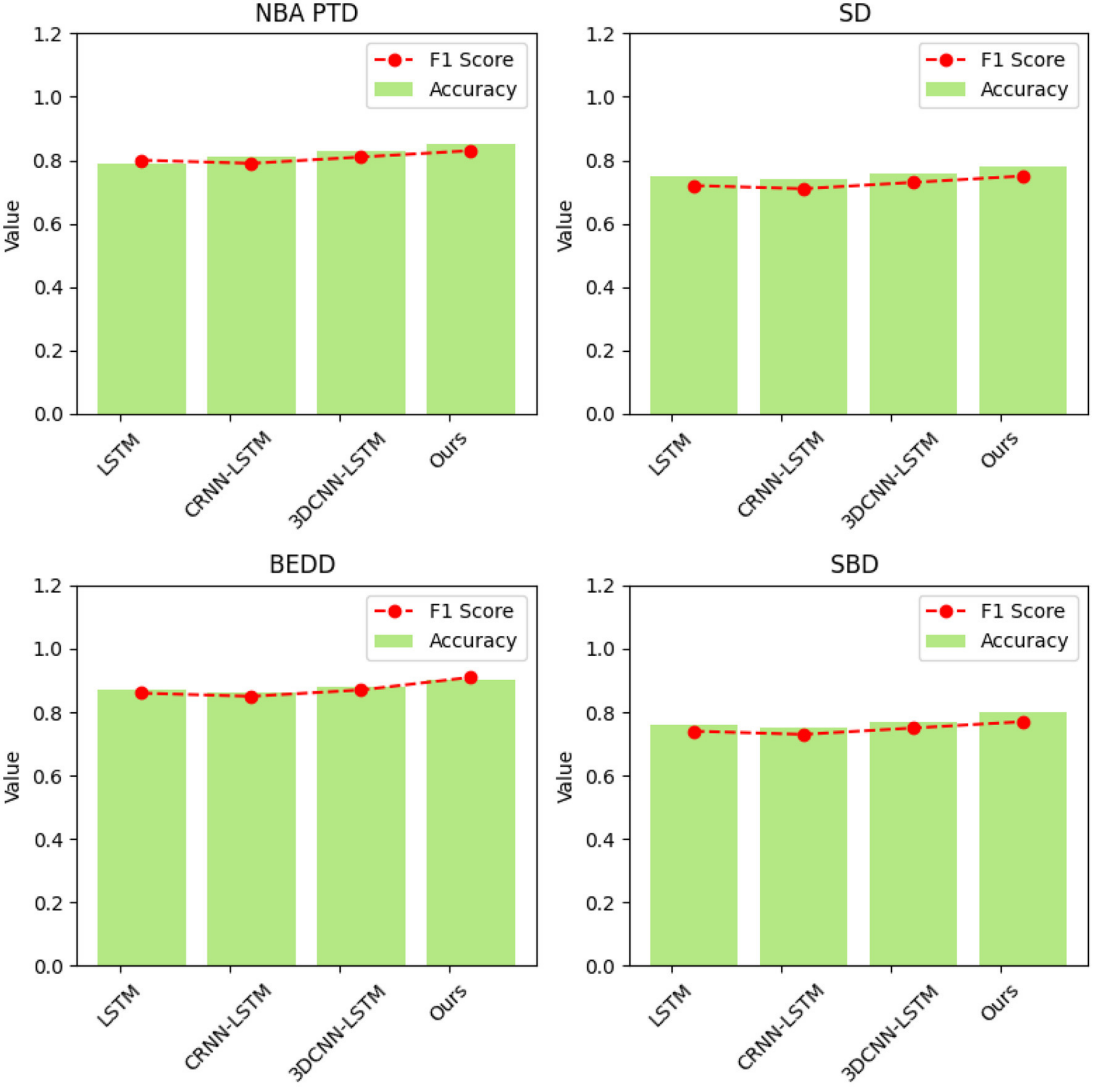
Comparative visualization of ablation experiments on NBA PTD, SD, BEDD, and SBD.

Table 5 and Figure 8 illustrate the results of our ablation experiments, which were designed to assess the influence of individual model components on accuracy and F1 Score metrics across four distinct datasets: NBA PTD, SD, BEDD, and SBD. These experiments aimed to uncover the specific contributions of each model component to the overall performance of our proposed “3DCNN-CRNN-LSTM Net” in recognizing and predicting player behaviors in basketball matches.

**Table 5:** Comparative visualization of ablation experiments of accuracy and F1 Score metric on four datasets.

**Model**	**Datasets**							
		**NBA PTD**		**SD**		**BEDD**		**SBD**	
		**Accuracy**	**F1 Score**	**Accuracy**	**F1 Score**	**Accuracy**	**F1 Score**	**Accuracy**	**F1 Score**
LSTM	0.79	0.80	0.75	0.72	0.87	0.86	0.76	0.74
CRNN-LSTM	0.81	0.79	0.74	0.71	0.86	0.85	0.75	0.73
3DCNN-LSTM	0.83	0.81	0.76	0.73	0.88	0.87	0.77	0.75
Ours	0.85	0.83	0.78	0.75	0.90	0.91	0.80	0.77

In our ablation experiments, we evaluated four different model configurations: LSTM, CRNN-LSTM, 3DCNN-LSTM, and our complete “Ours” model. The metrics used for comparison in Table 5 include Accuracy and F1 Score.

Analyzing the results, it becomes evident that our complete “Ours” model consistently outperforms the other configurations across all datasets. This underscores the synergistic and complementary nature of the three model components—3D CNN capturing spatial-temporal features, CRNN analyzing audio, and LSTM modeling sequential context. Together, these components create a comprehensive understanding of player behavior dynamics.

Our proposed “3DCNN-CRNN-LSTM Net” aligns seamlessly with the multimodal nature of basketball games by jointly analyzing visual and audio cues. This integration effectively captures intricate player actions and contextual information, addressing the inherent complexity of the task. As a result, our model achieves higher accuracy and precision in predicting player actions compared to the individual model components.

## 5 Conclusion and discussion

This article aims to solve the problem of player behavior recognition and prediction in basketball games. By fusing multi-modal audio-visual information, we propose a multi-modal audio-visual robot framework based on 3D CNN, CRNN and LSTM. By integrating three different deep learning components, 3D CNN, CRNN, and LSTM, the model can simultaneously extract rich features from video and audio information, and realize accurate recognition and prediction of player behavior in basketball games. The 3D CNN is used to capture the spatial and temporal information in the video, the CRNN analyzes the voice information, and the LSTM models the sequence information to comprehensively analyze the basketball game data. In order to fully explore and evaluate the model, we conducted a series of experiments to compare the performance of our method with other classical models under different datasets. It can be seen from the experimental results that our method performs well under multiple evaluation indicators, achieving higher accuracy, stability, and generalization performance. Especially in the comparative experiments on various data sets, our method has always maintained a leading position, not only achieved higher accuracy and F1 Score in the recognition task, but also showed better performance in the prediction task, revealing that Its efficacy and superiority in action recognition and prediction tasks.

However, this model also has some drawbacks: high computational complexity and large data requirements Since our model incorporates multiple deep learning components, the computational complexity of the model is high, requiring large computing resources and time for training and inference. Deep learning models usually require a large amount of data for training in order to achieve good generalization performance. In some cases, it may be difficult to obtain enough multimodal data, especially in specific scenarios or applications, it may be difficult to collect enough audiovisual information data. Future research can explore how to optimize the model structure and parameters to improve the computational efficiency of the model while maintaining the model performance. Using methods such as lightweight network structure or model pruning can reduce the demand for computing resources to a certain extent. In the future, technologies such as small sample learning and transfer learning can be considered to train models with limited data. The performance of models with limited data can be improved by transferring knowledge from other related domains or tasks.

This study proposes a deep learning method based on multimodal audio-visual data for the problem of player behavior recognition and prediction in basketball games. This model can help coaches and teams better understand the game process and player performance, so as to formulate more scientific tactics and decisions. It can also be used for in-depth analysis of game data, digging out information hidden behind the data, and providing fans and professional analysts with deeper insights.

## Data availability statement

The original contributions presented in the study are included in the article/supplementary material, further inquiries can be directed to the corresponding author.

## Author contributions

HW: Funding acquisition, Investigation, Methodology, Project administration, Resources, Software, Supervision, Validation, Writing—review & editing.
